# Research progress on perioperative blood-brain barrier damage and its potential mechanism

**DOI:** 10.3389/fcell.2023.1174043

**Published:** 2023-04-10

**Authors:** Yong Qiu, Chunheng Mo, Shiyu Xu, Lu Chen, Wanlin Ye, Yi Kang, Guo Chen, Tao Zhu

**Affiliations:** ^1^ Department of Anesthesiology, National Clinical Research Center for Geriatrics and The Research Units of West China (2018RU012), West China Hospital, Sichuan University, Chengdu, China; ^2^ Laboratory of Anesthesia and Critical Care Medicine, National-Local Joint Engineering Research Center of Translational Medicine of Anesthesiology, West China Hospital, Sichuan University, Chengdu, China; ^3^ Key Laboratory of Birth Defects and Related Diseases of Women and Children of MOE, State Key Laboratory of Biotherapy, West China Second University Hospital, Sichuan University, Chengdu, China

**Keywords:** BBB, endothelial cells, neuroinflammation, oxidative stress, ferroptosis, endothelial dysfunction

## Abstract

The blood-brain barrier (BBB) is an important barrier separating the central nervous system from the periphery. The composition includes endothelial cells, pericytes, astrocytes, synapses and tight junction proteins. During the perioperative period, anesthesia and surgical operations are also a kind of stress to the body, which may be accompanied by blood-brain barrier damage and brain metabolism dysfunction. Perioperative blood-brain barrier destruction is closely associated with cognitive impairment and may increase the risk of postoperative mortality, which is not conducive to enhanced recovery after surgery. However, the potential pathophysiological process and specific mechanism of blood-brain barrier damage during the perioperative period have not been fully elucidated. Changes in blood-brain barrier permeability, inflammation and neuroinflammation, oxidative stress, ferroptosis, and intestinal dysbiosis may be involved in blood-brain barrier damage. We aim to review the research progress of perioperative blood-brain barrier damage and its potential adverse effects and potential molecular mechanisms, and provide ideas for the study of homeostasis maintenance of brain function and precision anesthesia.

## Introduction

The blood-brain barrier (BBB) is an important barrier separating the central nervous system from the periphery. It can protect the brain from harmful substances in the peripheral blood and block the entry of inflammatory factors, thereby maintaining the homeostasis of the brain. The composition of BBB neurovascular units includes endothelial cells, pericytes, astrocytes, synapses and tight junction proteins. In addition, there are a variety of ion channels, receptors and carrier-mediated transport (CMT) on the BBB endothelium and pericytes that mediate substance transport and information transmission, and there is close information crosstalk between the endothelium, pericytes and astrocytes (Zhao et al., 2015; Mäe et al., 2021). Homeostasis in the BBB is very important for maintaining normal brain function. BBB disruption is closely related to neurodegenerative diseases. By regulating BBB-related immune activation and Wnt ligands, BBB structure and function can be restored (Alajangi et al., 2022; Martin et al., 2022). Specifically, there are tight junctions (TJs), gap junctions (GJs), and adhesion junctions (AJs) between endothelial cells. TJs consist of claudin, occludin, and zonula occludens proteins. GJs consist of connexin30 and connexin43. AJs mainly consist of vascular endothelial cadherin. These proteins connect endothelial cells and limit the permeability. In addition, there is basement membrane between pericytes and astrocytes. Abnormalities in these proteins may lead to the BBB destruction, of which TJs has received much attention. Matrix metalloproteinase (MMP) and Wnt signaling play a crucial in affecting TJs and basement membrane, thereby regulating BBB permeability and homeostasis (Obermeier et al., 2013; Sweeney et al., 2019; Yang and Chen, 2022).

Postoperative delirium (POD) is defined as acute emergence of confusion, emotional dysregulation, and perceptual disturbances. POD may occur soon after anesthesia and surgery and is usually acute, transient. Postoperative cognitive dysfunction (POCD) is characterized by memory and perceptual function impairment and persisting for more than 30 days. POD and POCD may contribute to prolonged hospitalization, additional burden and poor long-term outcomes (Liu et al., 2022a). BBB damage may be related to complications such as POD and POCD, which suggests that perioperative BBB protection deserves more attention (Wang et al., 2017). During the perioperative period, anesthesia, and surgical operations are also a kind of stress to the body, which may be accompanied by BBB damage and brain metabolism disorder. Moreover, the cerebrospinal fluid/plasma albumin ratio (CPAR) and plasma S100B are biomarkers for evaluating BBB permeability and BBB damage. Perioperative CPAR and plasma S100B increased significantly in patients with POD, suggesting the destruction of the BBB during the perioperative, and the severity of POD was related to BBB damage (Taylor et al., 2022). Furthermore, animal experiments have also found that surgery can cause damage to the BBB ultrastructure and changes in permeability, accompanied by cognitive impairment (He et al., 2012). Suo et al. (2022) used sevoflurane to anesthetize mice to construct a model of cognitive impairment. Then, the hippocampus was extracted and sequenced to identify functional pathways related to BBB damage, which further suggested that anesthesia and surgical exposure could potentially disrupt the BBB.

However, the potential pathophysiological process and specific mechanism of BBB damage have not been fully elucidated. Dysfunction of the vascular system has been implicated in aging and cognitive diseases. Elderly and cognitive decline individuals are more prone to arteriosclerosis and vascular system dysfunction. The integrity of the BBB in these individuals is usually destroyed, accompanied by inflammatory infiltration, glial cell activation, and Aβ accumulation. Therefore, whether targeted regulation of endothelial function can restore BBB homeostasis in the elderly and relieve cognitive impairment is worth exploring (Wang et al., 2020; Bhatia et al., 2021; Fisher et al., 2022). In addition, these individuals may be more susceptible to perioperative anesthesia and surgical intervention. The perioperative protection of brain function in aging deserves attention.

We aim to review the research progress of perioperative BBB damage and its potential effects and potential molecular mechanisms, and provide ideas for the study of homeostasis maintenance of brain function and precision anesthesia.

## Focus on perioperative BBB dysfunction

### Perioperative factors associated with BBB damage

BBB homeostasis is a process of dynamic regulation, and there are many factors that may potentially disrupt the BBB during the perioperative period. Analysis of the different brain temperatures of rats under anesthesia found that the brain temperature is an important factor in regulating BBB permeability, suggesting the potential influence of perioperative temperature on the BBB (Kiyatkin and Sharma, 2009). In addition, perioperative pain may cause glial cell activation, which in turn promotes the release of substance P and inflammatory factors to aggravate BBB damage (DosSantos et al., 2014). POCD may be related to postoperative opioid consumption in fast-track arthroplasty patients, suggesting that opioids exposure may also be associated with cognition decline and BBB damage (Awada et al., 2019). Patients with aging and neurodegenerative diseases have abnormalities in the BBB. As a result, they are more sensitive to perioperative stimulation and more prone to BBB damage (Sweeney et al., 2018). Anesthesia and surgical exposure are also a kind of stress, and their impact on the structure and function of the BBB has attracted much attention.

### BBB damage caused by anesthesia

Cao et al. (2015) anesthetized elderly rats with isoflurane and analyzed the ultrastructure of the hippocampal BBB by transmission electron microscopy at different time points after treatment. The expression of tight junction proteins and BBB permeability were detected. Isoflurane anesthesia caused reversible time-dependent ultrastructural morphological damage to the BBB, and the expression of occludin was significantly reduced, accompanied by leakage of the dye, proving that isoflurane exposure caused damage to the hippocampal BBB in elderly rats. Similarly, Acharya et al. (2015) found that sevoflurane significantly increased BBB permeability in elderly rats. Electron microscopy revealed changes in the BBB endothelium and tight junction structure, confirming the potential adverse effects of sevoflurane on BBB structure and function. Moreover, the potential effects of the intravenous anesthetic propofol have also attracted attention. After newborn mice were given propofol, transmission electron microscopy confirmed the dose-related microstructure changes in the cortex and the penetration of the dye at the same time. Molecular testing found abnormal expression of heat shock protein 72 molecules in the cerebral cortex and hippocampus, suggesting that propofol may induce cell stress to disrupt BBB integrity and then damage brain function (Sharma et al., 2014). Endothelium is an important component of BBB. *In vitro* experiments have also found that propofol stimulates brain endothelial cells and upregulates inflammatory factors and chemokines. The anti-inflammatory agent febuxotan can reduce oxidative stress and the inflammatory response and relieve the cytotoxic effects of propofol on brain endothelial cells (Hao et al., 2021). In addition, Hughes et al. (2022) used propofol to intervene in a BBB model derived from human induced pluripotent stem cells, and found that transendothelial resistance decreased and permeability increased, accompanied by occludin and matrix metalloproteinase (MMP) dysregulation, which provided evidence and ideas for anesthetic drugs to disrupt the BBB. However, the differences and specific mechanisms of BBB damage caused by different anesthetic drugs need further research.

### Surgery aggravates BBB damage

Surgery is inseparable from anesthesia, and the damage to the BBB caused by anesthesia and surgical intervention is often more significant than that of simple anesthesia exposure. Danielson et al. (2020) included 34 joint replacement subjects for preliminary analysis of serum and cerebrospinal fluid inflammation and BBB integrity biomarkers before and 48 h after surgery and evaluated cognitive function. The results showed that immune-related factors such as IL6 and CXCL6 in cerebrospinal fluid were significantly elevated in patients with poor cognition. The markers of BBB damage, CPAR and S100B, also increased transiently after surgery, which provided evidence for the destruction of the BBB and the emergence of neuroinflammation (Danielson et al., 2020). There is no doubt that surgery is closely related to the increase in inflammatory biomarkers in cerebrospinal fluid and blood. A recent study evaluated the changes in plasma and cerebrospinal fluid proteins after orthopedic surgery in elderly patients. It was also found that 343 proteins were significantly upregulated or downregulated 1 day after surgery compared to before surgery. Compared with 1 month after surgery, 67 proteins in plasma and 79 protein levels in cerebrospinal fluid were still significantly changed. Functional analysis suggested that many of these proteins are involved in inflammation and immunomodulatory processes, including multiple molecules that regulate the Wnt signaling pathway (Dillon et al., 2023). In addition, Reinsfelt et al. (2012) collected cerebrospinal fluid from subjects undergoing extracorporeal circulation heart surgery the day before and 24 h after the operation to assess glial cell damage, BBB integrity, and cytokine levels. The results showed that S100B, interleukin-6 and interleukin-8 increased significantly, and the CPAR ratio increased by 61%, suggesting glial cell damage and BBB dysfunction (Reinsfelt et al., 2012). Animal experiments also found evidence of BBB damage after surgery. Yang et al. (2017) found that the BBB permeability increased significantly after isoflurane anesthesia combined with cesarean section, accompanied by the downregulation of proteins claudin and occludin, with more significant changes in older mice. Paradoxically, another study that included 29 elderly patients with joint surgery under spinal anesthesia found that interleukin-6 and C-reactive protein (CRP) increased significantly 1 month after surgery compared with baseline, but there was no significant change in CPAR, suggesting that the duration and confounding factors of BBB damage after surgery need to be explored in depth (Vasunilashorn et al., 2021). We summarized the surgical method, sampling position, and sampling time points in Supplementary Table S1.

## Potential pathophysiological processes and mechanisms

### Increased BBB permeability

The BBB is a dynamically balanced barrier, and the typical change in the BBB in the perioperative period is increased permeability. Zhao et al. (2014) exposed human cerebrovascular endothelial cells to isoflurane and found that hypoxia-inducing factor-1α (HIF-1α) in endothelial cells was upregulated, while the expression of occludin was downregulated. This suggests that isoflurane may upregulate HIF-1α and mediate the downregulation of tight junction proteins, thereby destroying the BBB interface structure and increasing permeability (Zhao et al., 2014). Similarly, Cao et al. (2018) exposed elderly rats to isoflurane and detected downregulation of tight junction proteins between the cerebrovascular endothelium and increased permeability. Further studies confirmed that isoflurane exposure upregulates HIF-1α in the hippocampus, while inhibiting HIF-1α can improve downstream kinase activation, thereby relieving BBB damage and cognitive decline (Cao et al., 2018). The tight junction of the endothelium is an important structure that maintains the BBB barrier, and it is sensitive to changes in the extracellular matrix microenvironment. MMP3 knockout mice exuded less dye after exposure to isoflurane. Endothelial cell experiments confirmed that the upregulation of MMP3 destroys the integrity of the inter-endothelial barrier, while inhibiting MMP3 can increase the abundance of tight junction proteins (Zhang et al., 2021). In addition, endothelial cells and pericytes play an important role in the maintenance of BBB permeability. The unc5b receptor of BBB endothelial cells and its ligand Netrin-1 can regulate the integrity of the BBB through the Wnt/β-catenin signaling pathway (Boyé et al., 2022). Furthermore, there is close information crosstalk among pericytes, endothelial cells and astrocytes. Meanwhile, pericytes can regulate endothelial cell endocytosis. Therefore, pericyte dysfunction may lead to changes in permeability and BBB disruption (Zheng et al., 2020). By using BBB models and animal experiments, Yang et al. (2023) confirmed that omega-3 fatty acids can regulate endothelial structure and permeability. Omega-3 fatty acid supplementation can relieve pericyte dysfunction and BBB damage (Yang et al., 2023). In general, the specific effects of perioperative anesthesia and surgical exposure on endothelial cells and pericytes, as well as the specific process and molecular mechanism of BBB permeability changes, have yet to be clarified.

### Inflammation and neuroinflammation

Perioperative anesthesia and surgical exposure can cause peripheral inflammation and neuroinflammation, which in turn affect the integrity of BBB structure and function, and are closely associated with permeability changes. The level of peripheral and central inflammation increased in mice after receiving isoflurane anesthesia and internal fixation of tibial fractures, accompanied by BBB damage. Interestingly, inhibiting peripheral interleukin-6 levels in advance may relieve BBB destruction and hippocampal glial cell activation caused by anesthesia and surgical exposure (Hu et al., 2018). Versele et al., 2022 used endothelial cells and pericytes to construct a BBB cell model and intervened with TNF-α (tumor necrosis factor α) and IL-1β. Then, they found that inflammatory factors may change the interface between endothelial cells and affect the transport of paracellular substances, providing evidence for the destruction of the BBB by peripheral inflammatory factors (Versele et al., 2022). In addition, in some patients with an inflammatory state, central glial cell activation can secrete proinflammatory factors that directly act on the endothelial structure, while secreting chemokines to attract peripheral inflammatory cells infiltration and release the inflammatory factors IL-6, IL-1β, and TNFα. As a result, multiple inflammatory pathways are activated in a cascade, which ultimately leads to the destruction of BBB structure and function (Yang et al., 2022a).

Consistent with this, Liu et al. (2021) treated elderly mice with the anti-inflammatory agent atorvastatin and found that atorvastatin improved the increase of inflammatory factors in the hippocampus, and downregulated the nuclear factor kappa-beta inflammatory pathway. At the same time, the endothelial connection structure was repaired, and BBB damage was relieved (Liu et al., 2021). However, a randomized controlled trial analyzed the effects of glucocorticoid methylprednisolone supplementation on systemic inflammation and neuroinflammation after heart surgery. The results showed that the hormone significantly inhibited peripheral inflammation, but CPAR and serum S100B did not significantly improve. This shows that methylprednisolone does not reduce neuroinflammation and BBB damage, suggesting that follow-up studies need to further focus on neuroinflammation (Danielson et al., 2018).

### Oxidative stress and ferroptosis

**FIGURE 1:**
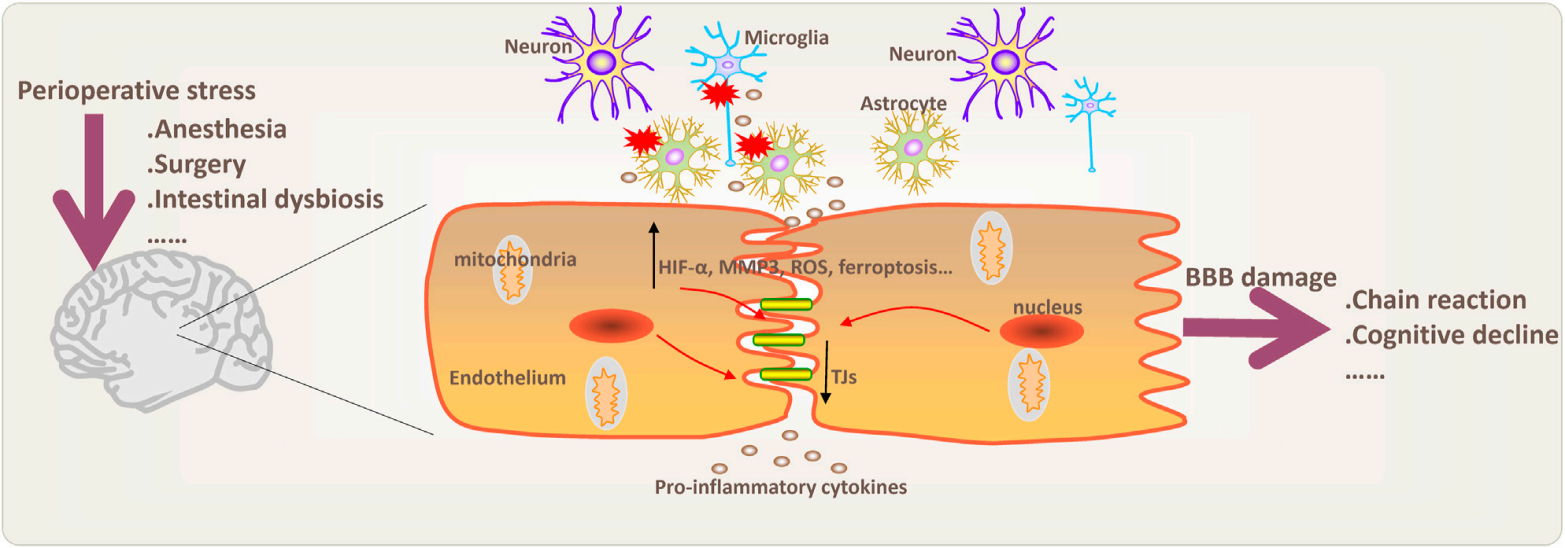
Potential pathophysiological processes and mechanisms involved in BBB damage during perioperative period. Changes in blood-brain barrier (BBB) permeability, inflammation and neuroinflammation, oxidative stress, ferroptosis, and intestinal dysbiosis may be involved in BBB damage. Chain reaction mean that BBB damage may cause peripheral inflammatory cell infiltration into the brain and further aggravate neuroinflammation. Moreover, activation of microglia and astrocytes may play an important role in the regulation of anxiety, depression and chronic neurodegenerative diseases. MMP3 (matrix metalloproteinase 3); ROS (reactive oxygen species); TJs (tight junctions).

Mitochondrial oxidative stress also plays an important role in BBB damage. Reactive oxygen species (ROS) may mediate mitochondrial stress and are closely associated with tight junction protein expression and MMP activation. Liu et al. (2022b) found that ROS and MMP were abnormally activated after anesthesia and surgical exposure in elderly mice, and ROS could regulate chemokine leukocyte factors to destroy the BBB structure. Eliminating ROS can reverse BBB damage, while synaptic plasticity and POD are improved, which provides evidence for oxidative stress-mediated perioperative BBB damage (Liu et al., 2022b). Similarly, Lee et al. (2022) isolated endothelial cells to delve into the role of endothelial cell mitochondria in BBB damage. Then, they found that endothelial Notch1 signaling was downregulated and BBB permeability increased in mice. At the same time, activating the Notch1 pathway could relieve BBB damage caused by endothelial mitochondrial dysfunction. Moreover, perioperative inhalation and intravenous anesthetic exposure may inhibit the electron transport chain complex and thus affect mitochondrial function. Therefore, it can be speculated that brain mitochondrial dysfunction during the perioperative period is closely associated with BBB damage (Fedorov et al., 2023). Oxidative stress is also closely related to neuroinflammation. The use of dexmedetomidine during the perioperative period can inhibit the activation of glial cells in the hippocampus, and can enhance superoxide dismutase to improve oxidative stress, providing evidence that dexmedetomidine may improve cognitive impairment through its anti-inflammatory and antioxidant properties (Xie et al., 2021). In addition, when the glutathione-dependent lipid peroxide repair system is damaged, ROS accumulation can lead to iron-dependent forms of ferroptosis. As the center of cell energy metabolism, mitochondria damage is closely linked to lipid peroxidation and iron disorders. In addition, ferroptosis may play an important role in tight junction protein expression, inflammatory cell infiltration, and environmental homeostasis in the BBB (Chen et al., 2022; Zhao et al., 2023). Consistent with this, in the study of Fang et al., glutathione peroxidase 4 (GPX4) in microvascular endothelial cells after traumatic brain injury was significantly downregulated. Ferroptosis inhibitors can improve BBB permeability and endothelial cell injury. (Fang et al., 2022). The above research provides clues and ideas for the involvement of mitochondrial oxidative stress and ferroptosis in mediating BBB damage (Figure 1).

### Perioperative intestinal dysbiosis and others

The potential role of the intestinal dysbiosis in perioperative has also attracted much attention. Disorders of the gut microbiome during the perioperative, as well as the involvement of neurotransmitters and short fatty acids, lead to BBB damage. Supplementation with probiotics can increase the expression of tight junction proteins between endothelial cells and reduce BBB permeability, thereby relieving cognitive decline. Moreover, probiotic supplementation may also regulate synaptic function and glial cell activation, which provides ideas for regulating the gut-brain axis to protect the BBB (Yang et al., 2020a; Wen et al., 2020). Luo et al. (2021) explored the regulation of BBB in elderly mice by supplementation with cefazolin during the perioperative. The analysis found that cefazolin affected the levels of intestinal bacteria and short-chain fatty acids, while the endothelial structure was repaired and cognitive function improved (Luo et al., 2021). In addition, the role of small molecules such as genes and non-coding RNAs in regulating BBB permeability, neuroinflammation, oxidative stress, and perioperative cognitive impairment has recently received much attention. Li et al. (2019) analyzed the gene expression of hippocampal tissue by constructing a POCD mouse model. Then, they identified multiple differentially expressed genes (e.g., Smad7, Vrk2). Gene Ontology (GO) and Kyoto Encylopaedia of Genes and Genomes (KEGG) functional enrichment showed significant changes in neuroinflammation and metabolic pathways. These findings provide candidate genes and clues for the study of the molecular mechanism of perioperative BBB impairment and cognitive decline. Moreover, microRNA-572 mediates the expression of neural cell adhesion molecule 1 in hippocampal neurons, which in turn may participate in POCD (Yu et al., 2015). Wei et al. (2021) found that lncRNA NONMMUT055714 was downregulated in the hippocampus of POCD mice. Overexpression of NONMMUT055714 can regulate miR-7684-5p, oxidative stress, and relieve POCD. Similarly, Wu et al. (2021) found that the hippocampus of elderly mice with POCD differentially expressed 124 circRNAs. These molecules may mediate adhesion junction, ErbB and MAPK signaling pathway, and may mediate hippocampal inflammation by regulating Egfr and Prkacb (Wu et al., 2021). CircRNA may also play important role in maintaining BBB integrity. Recently, Yang et al. (2022b); Yang et al. (2022c) found that circ-FoxO3 can inhibit the mTORC1 pathway during ischemia/reperfusion and then regulate autophagy to protect BBB. Collectively, the specific molecular mechanism of non-coding RNA mediating the prevention and treatment of POCD is still worth exploring in depth.

Furthermore, new structural discoveries in the field of brain anatomy also provide new ideas and inspiration for the study of BBB damage. The glymphatic system is a newly discovered glial-dependent peripheral blood vessel network that clears harmful metabolites from the brain. When it is dysfunctional, neuroinflammation may be induced. Therefore, it can be speculated that the structural and functional changes in the glymphatic system during the perioperative period may be related to BBB damage and cognitive changes and are worth exploring (Ren et al., 2021). Similarly, it was recently reported that there is a fourth meningeal layer, the subarachnoid lymphatic-like membrane (SLYM), that separates the space of the subarachnoid cavity in the brains of mice and humans. SLIM has a structure similar to the mesothelium, is closely connected to the endothelium of cerebrovascular vessels and regulates the exchange of substances between cerebrospinal fluid and veins. It is worth noting that the number of immune cells increases in the immune activation state, suggesting that SLYM may play a role in regulating brain immune homeostasis (Møllgård et al., 2023). Overall, there are still many questions about the influence of special structural and functional changes on BBB homeostasis during the perioperative period that have yet to be explained. Whether intestinal-brain axis regulation is involved in this process remains to be explored.

## Potential adverse effects caused by BBB damage

### Perioperative neurocognitive disorders

Perioperative BBB damage is closely associated with changes in cognitive function. Bowman et al. collected cerebrospinal fluid and serum inflammation markers in elderly individuals and found that the BBB damage rate was 13.5%, and there was a correlation between BBB damage and cognitive decline. In addition, cerebrospinal fluid vascular endothelial growth factor, chemokines, and inflammatory factors may predict BBB damage (Bowman et al., 2018). Elderly patients are prone to BBB damage during the perioperative period. A clinical study evaluated the effects of propofol and sevoflurane exposure on POD in elderly patients over the age of 60. A total of 209 joint replacement participants were included. The results showed that the incidence of POD in the propofol anesthesia group was 33.0%, and that in the sevoflurane group was 23.3%, suggesting that the risk of POD occurrence after general anesthesia in the elderly is greater (Mei et al., 2020). What is more, Quan et al. (2019) included 120 elderly patients undergoing abdominal surgery under general anesthesia and divided them into shallow anesthesia and deep anesthesia groups based on the bispectral index. Then, it was found that the incidence of POCD in the deep anesthesia group was 19.2% 7 days after surgery, and 39.6% in the other group, and the plasma CRP and IL-1ß levels in the deep anesthesia group were significantly lower. In addition, the incidence of POCD in the two groups 3 months after surgery was still 10%–15% (Quan et al., 2019). POD and POCD caused by general anesthesia and surgery can affect the quality of life of elderly patients and prolong hospitalization.

Animal experiments also confirmed that BBB impairment mediates cognitive impairment. Huang et al. used surgical intervention in 18-month-old mice to establish a POCD model, combined with flow detection of the proportion of peripheral blood white blood cells, BBB permeability analysis, and monoclonal antibody consumption of peripheral neutrophils, and found that MMP9 expression in plasma and hippocampus increased. It has also been proven that MMP9 secreted by peripheral neutrophils may be involved in BBB damage mediating cognitive impairment in elderly mice (Huang et al., 2022). On the basis of anesthesia exposure, surgical intervention aggravated BBB damage. Wang et al. (2017) analyzed the cognitive function, dye penetration, tight junction ultrastructural changes and occludin expression of elderly rats in the three groups of isoflurane exposure, anesthesia combined with splenectomy and sham control. Then, it was found that postoperative BBB damage was obvious and cognitive impairment occurred in the anesthesia combined with surgery group, further suggesting that postoperative cognitive impairment is closely associated with BBB impairment (Wang et al., 2017). Similarly, Ni et al. (2018) found that BBB damage after tibial fracture in elderly mice was accompanied by behavioral cognitive impairment. At the same time, it was found that increased IL-17A levels caused by surgery may increase BBB permeability and are associated with abnormal expression of occludin and IL-17A receptors in the hippocampus. Interestingly, inhibiting IL-17A can relieve BBB damage while reversing cognitive damage (Ni et al., 2018).

### Chain reaction and may increase AD risk

BBB damage may cause peripheral inflammatory cell infiltration into the brain and further aggravate neuroinflammation. Moreover, activation of microglia and astrocytes not only participates in cognitive dysfunction but also plays an important role in the regulation of anxiety, depression and chronic neurodegenerative diseases. Therefore, neuroinflammation is not conducive to rapid recovery and may increase the risk of postoperative mortality and hospitalization costs. The potential adverse effects of brain glial cell activation caused by perioperative BBB damage on the pathophysiological processes of the body deserve attention (Yang et al., 2020b). In addition, a retrospective analysis study of some patients with central nervous system lymphoma who received repeated general anesthesia found that after repeated anesthesia exposures, computed tomography scans showed contrast agent spillage caused by BBB damage. At the same time, 13% of these subjects had focal epilepsy after surgery, 11.9% had nausea and vomiting, 7.6% had drowsiness, dullness, and some had headaches and other symptoms. This also reminds us that we need to pay more attention to the potential adverse effects of BBB damage on patients with brain trauma, combined diseases, and major surgery (Elkassabany et al., 2008). Furthermore, BBB disruption may independently predict the occurrence of Alzheimer’s disease (AD). Nation et al., 2019 used novel methods such as dynamic contrast-enhanced magnetic resonance imaging to study brain capillary damage. It was found that elderly participants with early cognitive dysfunction had pericyte injury, brain capillary damage and hippocampal BBB damage instead of atypical tau markers. At the same time, logical regression analysis suggested that BBB damage may predict the occurrence of cognitive impairment (Nation et al., 2019). Elderly patients are prone to abnormal blood vessel function and BBB damage, which in turn may be related to AD risk. This suggests that we need to pay more attention to the potential adverse effects of perioperative BBB damage on these individuals (Lin et al., 2021).

### Potential therapeutic targets and prospects

We have discussed that omega-3 fatty acid, IL-17A neutralizing antibody, probiotics, dexmedetomidine and atorvastatin supplementation may protect BBB by improve permeability or inflammation in the above. Specifically, Yang et al. (2023) confirmed that omega-3 fatty acids supplementation may relieve BBB damage in POD mice. Freund Levi et al. (2014) included AD participants and found that continuous oral administration of omega-3 fatty acid for 6 months can change the fatty acid profile of cerebrospinal fluid, thus affecting phosphorylated tau and inflammatory biomarkers. Moreover, Barnes et al. (2021) analyzed 45 healthy elderly individuals of omega-3, cognitive function, and BBB integrity. The correlation analysis suggested that there is a positive correlation between omega-3 levels and the BBB integrity, indicating the potential beneficial role of omega-3 in maintaining BBB homeostasis (Barnes et al., 2021). Dexmedetomidine may regulate glial cell activation and immune T cell polarization, thus relieving neuroinflammation and BBB damage in mice with sepsis (Tian et al., 2021). In addition, a systematic review showed that dexmedetomidine may reduce the incidence of POD in adult cardiac and non-cardiac surgery patients (Duan et al., 2018). Wang et al. (2022) found that dexmedetomidine may regulate the levels of serum S100B, cortisol, interleukin-6 and TNF-α, and improve the cognitive function scores in elderly patients after surgery. Clinically, the protection and potential benefits of omega-3 fatty acid and dexmedetomidine for perioperative BBB are worthy of further investigation.

Resveratrol and metformin can also regulate inflammation-related pathways to mediate glial cell activation and thereby improve perioperative neuroinflammation and cognitive impairment. Therefore, it can be speculated about its potential role in protecting BBB (Tang et al., 2021; Peng et al., 2022). The role of metformin in aging and cognitive protection has received attention. Metformin may regulate hippocampal neuroinflammation, microglia activation, tau phosphorylation, and NF-kB pathway to relieve cognitive impairment. Clinical studies have also suggested that metformin supplementation potentially reduces the risk of AD (Ou et al., 2018; Khezri et al., 2022). Further evidence is needed for the protective effect of metformin on perioperative BBB.

In addition, mind bomb-2 (MIB2) and ferroptosis inhibitor liproxstatin-1 may regulate iron death-related pathways and oxidative stress in the hippocampus, which in turn may repair perioperative BBB damage (Zhao et al., 2021; Li et al., 2022). Ferroptosis may be involved in the pathophysiological processes of AD. NADPH oxidase 4 (NOX4) regulates mitochondrial metabolism, reactive oxygen species (ROS), oxidative stress and mediates astrocyte ferroptosis thus participating in AD. Targeted regulation of ferroptosis may improve brain homeostasis in patients with neurodegenerative diseases and ischemic stroke, thus affecting the prognosis (Millán et al., 2021; Park et al., 2021). The potential benefit of regulating ferroptosis on perioperative BBB protection also requires more evidence.

Recently, single-cell sequencing analysis of the hippocampus after sevoflurane exposures in mice found that different types of cells differentially expressed multiple genes. At the same time, astrocytes endocytosis, microglia apelin signaling pathway, endothelial cells glutamatergic synapse are typically enriched, and AD-related enrichment occurs in hippocampal neurons (Song et al., 2023). This suggests that in-depth exploration of the key gene targets and pathways involved in perioperative BBB damage and personalized intervention have broad prospects, and there is still a lot of work to be done.

## Conclusion

Perioperative anesthesia and surgical exposure may cause BBB damage, which in turn is related to poor postoperative prognosis. The role of BBB permeability changes, inflammation and neuroinflammation, oxidative stress, ferroptosis, and the gut microbiome in perioperative BBB damage and molecular mechanisms need to be further explored. Perioperative BBB protection, brain function monitoring and homeostasis maintenance have important clinical value and deserve more attention, especially in elderly individuals.
