# Supplementary material for: Research progress on perioperative blood-brain barrier damage and its potential mechanism
Source: Front Cell Dev Biol. 2023 Apr 10;11:1174043. doi: 10.3389/fcell.2023.1174043 (PMC10124715; doi:10.3389/fcell.2023.1174043)
Supplement: Supplementary file 1 [file Table1.DOCX]

| Table S1. Detail information of CSF and plasma markers change. | | | |
| --- | --- | --- | --- |
| Anesthetic and surgical method | Sampling position and time points | Main findings | References |
| Non-intracranial surgery | CSF and plasma; CSF was only collected if a spinal drain was placed for clinical reasons for vascular surgery. During days 1-4, researchers obtained blood. Samples for CPAR (n=25) and plasma S100B (n=78). | There were greater increases in CPAR and plasma S100B in those with delirium. There is an association of delirium incidence with changes in both CPAR and S100B. | Taylor et al., 2022 |
| 34 patients scheduled for elective total hip or knee replacement surgery. Spinal anesthesia and supplemented with light intravenous propofol. | Serum and CSF. Preoperatively and at 4, 8, 24, 32 and 48 hours after skin incision. | CPAR and S100B increased transiently | Danielson et al., 2020 |
| Elective hip and knee replacement surgery with spinal anesthesia | Plasma and CSF proteins, blood plasma (n = 14) and CSF (n = 15) samples; preoperative, postoperative day1, and postoperative 1-month | 343 proteins were significantly upregulated or downregulated one day after surgery compared to before surgery. Compared with one month after surgery, 67 proteins in plasma and 79 protein levels in cerebrospinal fluid were still significantly changed. | Dillon et al., 2023 |
| 29 elderly patients with joint surgery under spinal anesthesia | Plasma and CSF samples collected at baseline and postoperative 1 month | plasma IL-6 increased,  no significant change in CSF/plasma albumin | Vasunilashorn et al., 2021 |
| Extracorporeal circulation heart surgery, general anesthesia with sevoflurane and propofol | CSF and blood sample, the day before and 24 hours after the operation | CSF S100B, IL-6 and IL-8 increased significantly, and the CPAR ratio increased by 61%. | Reinsfelt et al., 2012 |
| CSF: cerebrospinal fluid; CPAR: CSF/plasma albumin ratio; IL-6: interleukin-6; IL-8: interleukin-8. | | | |
